# Endemicity of Zoonotic Diseases in Pigs and Humans in Lowland and Upland Lao PDR: Identification of Socio-cultural Risk Factors

**DOI:** 10.1371/journal.pntd.0003913

**Published:** 2016-04-12

**Authors:** Hannah R. Holt, Phouth Inthavong, Boualam Khamlome, Kate Blaszak, Chattouphone Keokamphe, Virasack Somoulay, Anousone Phongmany, Peter A. Durr, Kerryne Graham, John Allen, Blánaid Donnelly, Stuart D. Blacksell, Fred Unger, Delia Grace, Silvia Alonso, Jeff Gilbert

**Affiliations:** 1 Department of Production and Population Health, Royal Veterinary College, London, United Kingdom; 2 National Animal Health Laboratory, Department of Livestock and Fisheries, Ministry of Agriculture and Forestry, Vientiane, Lao PDR; 3 Department of Communicable Diseases Control, Ministry of Health, Vientiane, Lao PDR; 4 Australian Animal Health Laboratory, CSIRO, Geelong, Australia; 5 World Animal Protection, Melbourne, Australia; 6 National Centre for Laboratory and Epidemiology, Vientiane, Lao PDR; 7 Veterinary Service Division, Department of Livestock and Fisheries, Ministry of Agriculture and Forestry, Vientiane, Lao PDR; 8 International Livestock Research Institute, Nairobi, Kenya; 9 Mahidol-Oxford Tropical Medicine Research Unit, Faculty of Tropical Medicine, Mahidol University, Bangkok, Thailand; 10 Centre for Tropical Medicine & Global Health, Nuffield Department of Clinical Medicine, University of Oxford, Oxford, United Kingdom; Jiangsu Institute of Parasitic Diseases, CHINA

## Abstract

In Lao People’s Democratic Republic pigs are kept in close contact with families. Human risk of infection with pig zoonoses arises from direct contact and consumption of unsafe pig products. This cross-sectional study was conducted in Luang Prabang (north) and Savannakhet (central-south) Provinces. A total of 59 villages, 895 humans and 647 pigs were sampled and serologically tested for zoonotic pathogens including: hepatitis E virus (HEV), Japanese encephalitis virus (JEV) and *Trichinella spiralis*; In addition, human sera were tested for *Taenia* spp. and cysticercosis. Seroprevalence of zoonotic pathogens in humans was high for HEV (Luang Prabang: 48.6%, Savannakhet: 77.7%) and *T*. *spiralis* (Luang Prabang: 59.0%, Savannakhet: 40.5%), and lower for JEV (around 5%), *Taenia* spp. (around 3%) and cysticercosis (Luang Prabang: 6.1, Savannakhet 1.5%). Multiple correspondence analysis and hierarchical clustering of principal components was performed on descriptive data of human hygiene practices, contact with pigs and consumption of pork products. Three clusters were identified: Cluster 1 had low pig contact and good hygiene practices, but had higher risk of *T*. *spiralis*. Most people in cluster 2 were involved in pig slaughter (83.7%), handled raw meat or offal (99.4%) and consumed raw pigs’ blood (76.4%). Compared to cluster 1, cluster 2 had increased odds of testing seropositive for HEV and JEV. Cluster 3 had the lowest sanitation access and had the highest risk of HEV, cysticercosis and *Taenia* spp. Farmers which kept their pigs tethered (as opposed to penned) and disposed of manure in water sources had 0.85 (95% CI: 0.18 to 0.91) and 2.39 (95% CI: 1.07 to 5.34) times the odds of having pigs test seropositive for HEV, respectively. The results have been used to identify entry-points for intervention and management strategies to reduce disease exposure in humans and pigs, informing control activities in a cysticercosis hyper-endemic village.

## Author Summary

In Lao PDR, pigs are an important source of food and income and are kept by many rural residents. This study investigated five diseases that are transmitted between pigs and humans (zoonoses), namely hepatitis E, Japanese encephalitis, trichinellosis, cysticercosis and taeniasis. Humans and pigs in Lao PDR were tested for antibodies against the agents (pathogens) responsible for these diseases. Human participants were classified into three groups or "clusters" based on hygiene and sanitation practices, pig contact and pork consumption. Cluster 1 had low pig contact and good hygiene practice. Cluster 2 had moderate hygiene practices: around half used toilets and protected water sources; most people washed their hands after using the toilet and boiled water prior to consumption. Most people in this cluster were involved in pig slaughtering, drank pigs’ blood and were more likely test positive for antibodies against hepatitis E and Japanese encephalitis viruses. Finally, people in cluster 3 had lowest access to sanitation facilities, were most likely to have pigs in the household and had the highest risk of hepatitis E, taeniasis and cysticercosis. The diseases in this study pose a significant threat to public health and impact pig production. This study identified characteristics of high-risk individuals and areas with high disease burden and could be used to target future disease control activities to those most vulnerable.

## Introduction

Approximately two thirds (66.9%) of the 6.4 million residents of Lao PDR reside in rural areas and most (83%) of the 0.8 million households are considered agricultural holdings [1]. The majority of these employ mixed farming systems (i.e. keeping both livestock and crops). In recent years, intensification of crop production has improved accessibility to remote villages which were previously isolated. Although this has many benefits for both crop and livestock production, e.g. improved access to markets, it also increases infectious disease transmission between villages. Historically, most pig-owning households employed traditional village practices (low-input, extensive scavenger systems), however farmers are switching to confined systems in order to reduce disease risk and prevent cash-crop damage [2]. Integrated pig production also occurs whereby pig faeces is utilized as an input for another production system such as manure for crops or fish feed.

Co-habitation with animals is common in Lao PDR; even in urban households and households where livestock rearing is not a major source of income [3]. Close proximity with livestock poses a risk of zoonotic infection via direct contact or environmental contamination. Additional potential transmission routes include consumption of unsafe products such as raw or undercooked pork, raw pig’s blood and fermented pork sausage. In Lao PDR, funding for human health care and veterinary services is lacking; resulting in poor access, low diagnostic capabilities and virtually non-existent surveillance and control of zoonotic diseases [4]. As a result, under-reporting of diseases is commonplace and public health and veterinary services’ capacity are readily overwhelmed by disease outbreaks [5].

The epidemiology of hepatitis E, cysticercosis/taeniasis, trichinellosis and Japanese encephalitis were investigated in this study. Stakeholders from the Ministry of Health, National Animal Health Laboratories and the National Centre for Laboratory and Epidemiology in Lao PDR, and previous research funded by the Australian Centre for International Agricultural Research (ACIAR) [6–9] identified these diseases as pig zoonoses of national importance.

Hepatitis E virus (HEV) is primarily water-borne and can cause acute hepatitis; transmission is via faecal-oral route and contaminated water is responsible for most outbreaks [10]. Symptoms include jaundice, abdominal pains, nausea and fever with high case fatality rate reported in pregnant women [10]. Zoonotic transmission occurs through consumption of undercooked contaminated meat and shellfish [11]. In addition, slaughterhouse workers, pig farmers and veterinarians have a high risk of occupational exposure [12]. Transmission routes for pigs are direct contact or ingestion of feed or water contaminated with faeces of infectious pigs. The disease in pigs is generally asymptomatic. Hepatitis E is generally endemic in regions with poor sanitation and hygiene including large parts of Asia. Previous estimates of HEV seroprevalence in pigs in Lao PDR in the Luang Prabang Province were 15% (dry season) and 47.1% (wet season) [7].

*Trichinella spiralis* is thought to be endemic in the pig population in Lao PDR and infection in humans occurs via the ingestion of raw or undercooked meat containing the larvae of *T*. *spiralis* nematodes [8, 13]. Suspected human cases occur regularly in Lao PDR, however, diagnostic facilities and outbreak investigation are lacking [5]. Large outbreaks of the disease usually occur at festivals or funerals and the largest reported outbreak in Lao PDR was in the north with 650 suspected human cases [5]. Transmission among pigs is through scavenging or feeding of undercooked meat containing the parasite. Faecal oral transmission and tail biting are believed to be minor routes of infection [14].

Japanese encephalitis, a vector-borne virus transmitted by *Culex* mosquitos is a major cause of morbidity and mortality in humans, and reduced productivity of pigs in Southeast Asia [15]. Epidemics occur after amplification of Japanese encephalitis virus (JEV) in immunologically naïve pigs housed close to human populations; most notably near rice paddies during the rainy season. A previous study in Oudomxay, Luangprabang, Xiengkhuang and Huaphan Provinces estimated the seroprevalence in pigs to be high (74.7%) [9].

*Taenia solium* causes human and porcine cysticercosis and is considered one of the most important diseases in Southeast Asia, and a neglected zoonotic disease [4]. Human taeniasis describes infection by the adult tapeworm following consumption of raw or undercooked pork contaminated with the larval stage of *T*. *solium* (or *T saginata* in beef) [16]. Cysticercosis in pigs and humans is caused by ingestion of *T*. *solium* eggs expelled from infected humans via food, water, or environmental faecal contamination. In humans, this can lead to the development of mature cysts in various organs including muscles, eyes, subcutaneous tissues and the central nervous system. Cysticercosis causes significant morbidity and mortality in humans and can lead to neuro-cysticercosis; the leading cause of epilepsy in the region [4]. Although, asymptomatic in pigs, losses occur due to the development of metacestodes leading to carcass condemnation. Previous prevalence estimates in Lao PDR (Vientiane) in pigs range from 0 to 14% [6].

The aim of the study was to estimate the seroprevalence of HEV, JEV, *T*. *spiralis* in humans and pigs and *Taenia* spp. and cysticercosis in humans in Luang Prabang (upland) and Savannakhet (lowland) Provinces and identify risk factors for infection. Focussing on ‘unsafe practices’ facilitates identification of entry points for intervention; providing useful information for the control and surveillance of zoonotic diseases in Lao PDR. These data are intended for use by animal and human health authorities to inform targeting of scarce resources to high risk populations.

## Methods

### Study area and population

The study was conducted in 2011 in one upland and one lowland Province of Lao PDR which differed in terms of climate, topography, farming systems, range of ethnicities and socioeconomic status. In addition to discussion with local partners, a report by the Swiss National Centre of Competence in Research (NCCR) which detailed geographic differences of indicators of socioeconomic status (e.g. sanitation, drinking water and education) was consulted to ensure variation in risk factors for the pathogens investigated [17].

Luang Prabang Province (20.21°N, 102.62°E), situated in northern Lao PDR covers an area of 16,875km^2^ and shares a border with Vietnam. At an altitude of 700 to 1,800m above sea level, it was selected to represent a typical upland Province. In addition to Lao Loum (the predominant ethnic group in Lao PDR) this Province is inhabited by Hmong (Lao Soung) who tend to reside in mountainous regions and Khmu (Lao Theung) who have settled at medium altitudes [18]. Each of these groups are unique in terms of culture, language, and differ in land-use practices and socio-economic status [18].

Savannakhet Province (26.54°N 105.78°S), situated in the southern-central part of the country shares a border with both Thailand and Vietnam, covers an area of 21,774km^2^ and is 145m above sea level. Lao Loum is the main ethnic group (>75%) with the remainder of the population being predominantly of Lao Theung ethnicity. The Province contains floodplains of the Mekong Delta and is the largest rice-producer in the country. Annual rainfall averages around 1,450mm per year and the Province is prone to both droughts and flooding [19]. Pig production is common and there are an increasing number of commercial pig farms close to the Thai border.

### Study design and sampling

The sample size calculation used a seroprevalence of 50% as little prior information was available and was sufficient to estimate human seroprevalence with 5% precision. In total, 59 villages were randomly selected (29 in Luang Prabang and 30 in Savannakhet) using probability proportional to human population. In each village, 15 households were randomly selected regardless of pig ownership during a village-wide meeting. Within these households, one household member over 5 years of age was randomly selected to be sampled and interviewed, resulting in a total of 895 human participants. A questionnaire for humans, developed in consultation with local health authorities, gathered information on socio-economic factors, pig-farming practices, cooking and eating behaviour, sanitation facilities and hygiene practices. Questionnaires were administered by district public health officials belonging to several ethnic groups and were conducted in native languages of the villagers.

Approximately 15 pig-owning households were randomly selected from each village. In each household, one pig over 12 weeks of age was randomly selected for blood sampling and the owner was interviewed. A questionnaire for pig owners gathered information on pig health and management. As sampling was done probability proportional to human size selected villages were found to have a range of pig densities, therefore the target of 15 pig-owning households could not be satisfied in all villages resulting in a total 647 pigs sampled. In addition, seroprevalence estimates for pigs will have lower accuracy and may be subject to bias. Therefore we will only refer to the percentage of pigs testing seropositive when discussing the pig results. Although the results will give an indication of the magnitude of the problem in pigs.

Knowledge dissemination to participating villages consisted of a summary of results and information regarding prevention of these diseases in pigs and humans. These sessions were carefully designed in an attempt to maximise the uptake of recommendations.

### Ethical consideration

Ethical approval was granted by the Institutional Research Ethics Committee (IREC) of the International Livestock Research Institute (ILRI) and the National Ethics Committee for Health Research in Lao PDR (No. 772 NIOPH/NECHR). All selected participants were asked to give informed written consent before being blood sampled and interviewed, if they were under the age of 18 then their parent or guardian provided consent and could give information on their behalf when needed. Owners of selected pigs were asked to give informed consent to be interviewed and for their pigs to be sampled.

### Serological survey of pathogens

All laboratory testing was performed in Lao PDR at the National Animal Health Laboratory, Ministry of Agriculture and Fisheries or the National Centre for Laboratory and Epidemiology, Ministry of Health. Blood samples were collected in plain vacutainers. Samples were refrigerated and then placed on ice until arrival at the laboratory, where they were stored at -20°C before testing.

Human serum samples were tested for the presence of antibodies against HEV, *T*. *spiralis*, JEV and the ratio of JEV to dengue virus antibodies using the following commercial diagnostic kits: HEV ELISA 4.0 (MP Diagnostics, Singapore, reported sensitivity of 98% and specificity of 96.7%), *T*. *spiralis* IgG ELISA (IBL International, Germany, reported sensitivity of 95% and specificity of 94.8%) and the JE-Dengue IgM Combo ELISA Test E-JED01C (Panbio, France, sensitivity at 89.3% and specificity at 99.2% using samples from Thailand [20]). Manufacturers’ instructions were followed when conducting and interpreting these kits. Antibodies against cysticercosis and *Taenia* spp. were detected using an enzyme-linked immunoelectrotransfer blot (EITB) as per Salim et al. (2009) [21]. This strip contains two recombinant antigens for cysticercosis (rT24H) and *Taenia* spp. (rES33). The detection of the T24 antigen has a sensitivity of 94% with two or more cysts in the brain [22], but drops to around 63% with only one cyst, specificity is around 98%. For *Taenia* spp. sensitivity of rES33 of 99.4% and specificity of 94.5% have been reported [23].

Pig serum samples were tested for the presence of antibodies against HEV using the HEV ELISA 4.0v kit (MP Diagnostics, Singapore: reported sensitivity of 98% and specificity of 96.7%); for *T*. *spiralis* antibodies using the Priocheck Trichinella Ab ELISA (Prionics, Switzerland. Sensitivity: 97.1–97.8% and specificity: 99.5–99.8%) [24]; and for JEV IgM specific antibodies and IgG specific antibodies using non-commercial ELISA kits developed by the Australian Animal Health Laboratory, Geelong Australia. Pigs were not tested for cysticercosis as part of this study as the antibodies lack diagnostic specificity and severe cross-reactivity can occur with pigs infected with other parasites (which may be present in the region). Manufacturers’ instructions were followed when using these kits.

### Statistical analysis

#### Data management

All questionnaire and serological data were entered into a custom-built web-based survey design and management application (“*SurVet*”). This application was designed with both English and Laotian language display features so that entry and data checking could be undertaken by a team member in their native language. The data were uploaded and stored in the *MySQL* relational database management system. Data cleaning and descriptive statistical analysis were conducted in Microsoft Excel. The remainder of statistical analyses were carried out in R (v. 3.0.1).

#### Exploratory data analysis

Seroprevalence of zoonotic pathogens were estimated for pigs and humans at the Province level and chi-squared tests were performed using the *stats* package to assess whether seroprevalence in humans or percentage of pigs seropositive differed significantly between Provinces.

Multiple correspondence analysis (MCA, a data reduction technique similar to factor analysis or principal component analysis) was applied to potential risk factors for human infection [25]. MCA was used to produce a graphic representation describing the relationships between the exposure variables. It also reduced the number of variables to analyse by creating uncorrelated synthetic dimensions which described the variation in the data with respect to human exposures [25]. Risk factor variables included in the analysis were: water sources, toilet access, pork consumption and food preparation habits (including consumption of fermented sausage and pigs’ blood) and contact with pigs including presence of pigs in the household, involvement in pig husbandry and participation in pig slaughtering.

General population characteristics e.g. gender, age and province were included as supplementary variables. This means they did not contribute to the creation of the dimensions but allowed the relationship between these variables and the dimensions and subsequent clusters to be described. The coordinates of each individual were calculated on the first 3 dimensions created by MCA and cluster analysis using hierarchical clustering on principal components (HCPC) was then performed on the coordinates of the selected dimensions using Ward’s method to aggregate individuals into relatively homogeneous subgroups (clusters). The analysis was performed using the package *FactoMineR*.

#### Risk factor analysis

Risk factor analysis was performed to assess whether membership of a particular cluster increased the risk of testing seropositive for any of the pathogens. Logistic regression models with random-effects were utilised with the three clusters identified from the MCA and HCPC as exposures and the serological status of the individual for each pathogen (HEV, JEV, *T*. *spiralis*, *Taenia* spp. and cysticercosis) as outcomes. Village was included as a random effect to control for the correlation of humans within villages. Age and gender were included as fixed effects to control for potential confounding effects of these variables. Gender was subsequently removed from any models where it was associated with the outcome with a *p*-value > 0.05.

Risk factor analysis was then performed with the serological results for HEV and *T*. *spiralis* in pigs. Chi-squared tests were used to assess the associations with variables of interest (housing, management, and herd and pig health) and the pathogens. Any variable associated with a pathogen with a *p*-value ≤ 0.2 were retained for further multivariable analysis. For each pathogen, random effects logistic regression models were used in order to identify associations between variables and the pathogen, controlling for potential confounding effects of other variables. A backwards step-wise elimination procedure was used and variables were removed from the models if the *p*-value was >0.05. The mixed effects logistic regression analyses for both humans and pigs was performed using the package *lme4*.

## Results

### Serological survey

In total 895 people and 647 pigs were sampled. Problematic samples (e.g. insufficient serum) or inconclusive test results were classified as missing (<10% for any pathogen). A high percentage of both pigs and people were seropositive for HEV (Table 1). However, humans were more likely to be seropositive in Savannakhet: 77.7% (95% Confidence interval (CI): CI: 73.7 to 81.6) vs. 48.6% (95% CI: 43.9 to 53.3), whilst pigs were more likely to be seropositive in Luang Prabang Province. There was a high seroprevalence of *T*. *spiralis* in humans; particularly in Luang Prabang Province (59.0%, 95% CI: 54.3 to 63.6). Seroprevalence for JEV in pigs was high; particularly in Savannakhet (81.4%, 95% CI: 76.8 to 85.8)

**Table 1:** Comparison of seroprevalence estimates (with 95% confidence intervals) for the selected of zoonotic pathogens in humans and percentage of pigs testing seropositive in Lao PDR, according to Province (*p-values refer to the results of chi-square tests).

Pathogen	Luang Prabang Province	Savannakhet Province	*p*-value
	% (95% CI)	% (95% CI)	
**Human results**			
Japanese encephalitis (N = 862)	4.9% (2.9% to 7.0%)	4.7% (2.8% to 6.9%)	0.96
Hepatitis E (N = 870)	48.6% (43.9% to 53.3%)	77.7% (73.7% to 81.6%)	<0.001
Trichinella (N = 822)	59.0% (54.3% to 63.6%)	40.5% (35.6% to 45.3%)	<0.001
Taenia (N = 844)	2.3% (0.9% to 3.7%)	2.9% (1.4% to 4.6%)	0.52
Cysticercosis (N = 826)	6.1% (3.9% to 8.4%)	1.5% (0.3% to 2.8%)	<0.001
**Pig results**			
Trichinella (N = 636)	14.4% (10.3% to 18.4%)	9.3% (6.2% to 12.4%)	0.05
Hepatitis E (N = 633)	81.3% (76.8% to 85.8%)	47.7% (42.4% to 53.0%)	<0.001
Japanese encephalitis (IgG) (N = 646)	73.0% (68.9% to 79.0%)	81.4% (76.8% to 85.8%)	0.02
Japanese encephalitis (IgM) (N = 627)	13.0% (7.8% to 18.2%)	7.1% (3.2% to 10.9%)	0.02

### Multiple correspondence & cluster analysis

**Fig 1:**
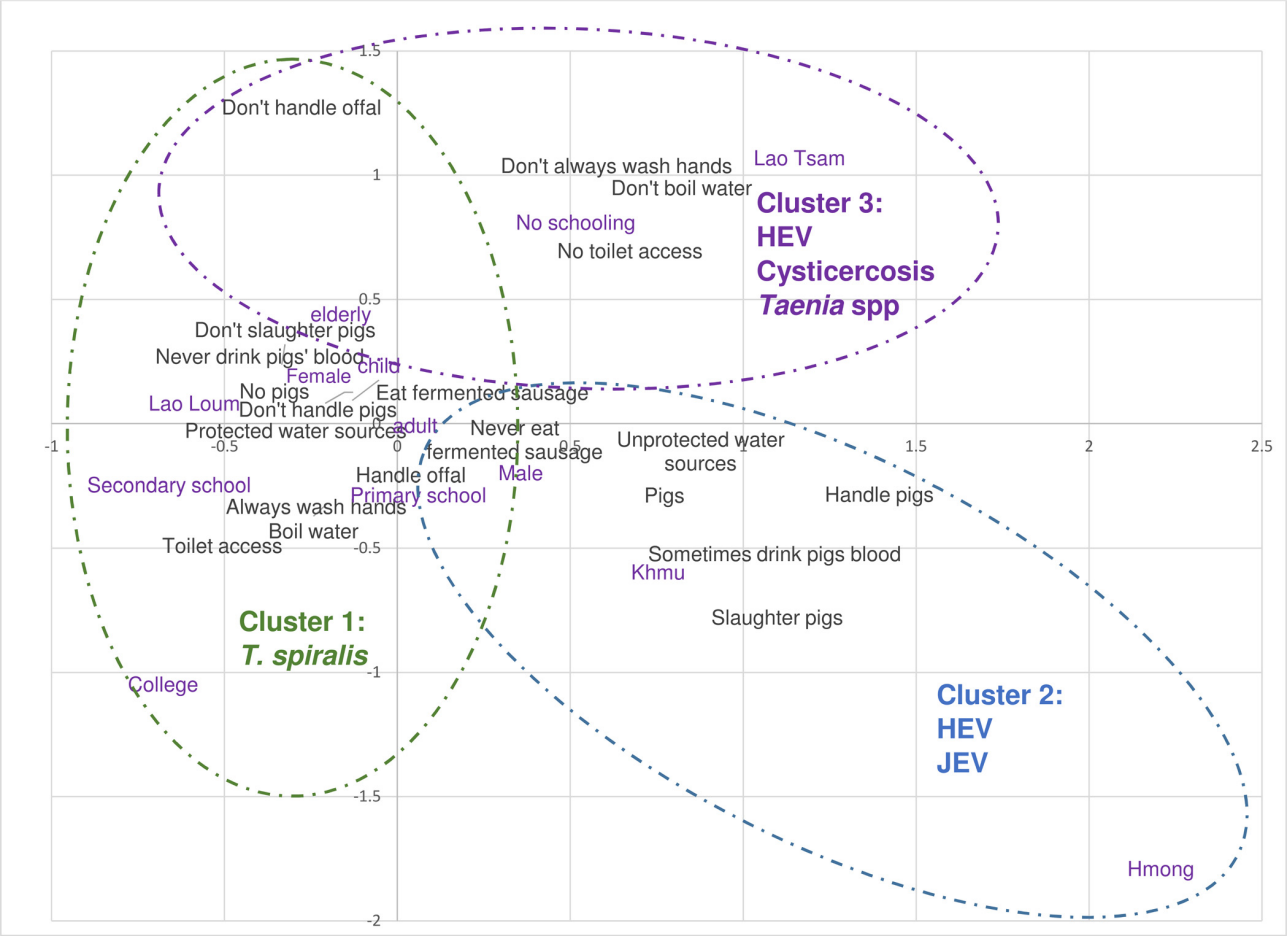
Summary of the results of MCA, HCA and risk factor analysis for pig zoonoses in humans in Lao PDR.

Fig 1 shows the coordinates of each variable on the two dimensions which explained the largest percentage of the variance in the data. Variables with coordinates close to zero are not well represented and the further away from the axis, the better represented the variable on that dimension. Variables which are closest to each other on the scatterplot are the most closely related. The variables in black are those which contributed to the creation of the dimensions; variables such as slaughtering pigs, pigs’ blood consumption and whether they boiled water before consumption are well represented on both dimensions. Type of water source and whether individuals handle pigs are better represented on dimension one (horizontal axis on Fig 1), whilst handling offal is well represented on the second dimension. Supplementary variables (purple) are also included on the scatterplot to visualise how these relate with the dimensions.

The cluster analysis was performed using the first three dimensions which explained 49.8% of the total variation and not less than 12% individually. The profiles of each cluster identified through HCA are described in Table 2; most people were classified as cluster 1 (51.1%). In general, this cluster had more females (65.6%), people were mainly Lao Loum (84.4%) and appeared to be better educated than the other clusters. They also appeared to have better hygiene practices with most people having toilet access (86.1%), washing their hands after the toilet (92.5%), using protected water sources (90.4%) and boiling water before consumption (92.1%). In terms of pig contact, most had no pigs in the household (83.0%) and did not handle or slaughter pigs (>95%). This cluster was used as the baseline for risk factor analysis.

**Table 2:** Description of the three typologies identified using MCA and HCA and allocation of all surveyed individuals, N = 895 (* denotes categories which exhibited differences between clusters with a p-value ≤ 0.05).

Variable	Cluster 1	Cluster 2	Cluster 3
	N = 458	N = 185	N = 252
**Demographic profile (supplementary variables)**			
Sex			
Male	34.3%*	83.1%*	49.2%
Female	65.7%*	16.9%*	50.8%
Age			
Children (≤16)	8.3%	5.1%*	8.1%
Adult (17 to 64)	68.2%*	80.3%*	79.2%*
Elderly (65+)	11.0%	4.5%	10.6%
Missing	12.5%*	10.1%	2.1%*
Ethnicity			
Hmong	0.4%*	14.0%*	0.8%*
Khmu	13.5%*	42.7%*	18.6%
Lao Loum	84.4%*	38.8%	59.7%*
Lao Tsam	0.8%*	2.8%	6.4%*
Laotheng	0.4%*	0.6%	6.8%*
Missing	0.4%*	1.1%	7.6%*
Province			
Luang Prabang	58.8%*	70.2%*	16.1%*
Savannakhet	41.2%*	29.8%*	83.9%*
Education			
No schooling	17.9%*	16.9%	42.4%*
Primary school	34.5%*	16.9%*	41.5%
Secondary school	43.7%*	54.5%*	16.1%*
College or University	4.0%*	1.7%	0%*
**Hygienic practices**			
Toilet use			
Yes	86.1%*	56.2%	7.2%
No	13.9%*	43.8%	92.8%*
Always wash hands after toilet			
Yes	92.5%*	83.1%*	38.1%*
No	7.5%*	16.9%*	61.9%*
**Drinking water**			
Unprotected water source			
Yes	9.6%*	48.9%*	34.7%*
No	90.4%*	51.1%*	65.3%*
Boil before consumption			
Yes	92.1%*	87.1%*	33.1%*
No	7.9%*	12.9%*	66.9%*
**Pig contact**			
Pigs in the household			
Yes	17.0%*	36.0%	64.0%
No	83.0%*	64.0%*	36.0%
Handle pigs			
Yes	3.3%*	39.3%*	19.1%*
No	96.7%*	60.7%*	80.9%*
Slaughter pigs			
Yes	5.4%*	83.7%*	15.4%*
No	94.6%*	16.3%*	85.6%*
Drink pigs blood			
Yes	10.6%*	76.4%*	23.3%
No	89.4%*	23.6%*	76.7%
Consume fermented sausage			
Yes	57.0%*	29.8%*	51.3%
No	43.0%*	70.2%*	48.7%
Handle offal/raw meat			
Yes	88.6%	99.4%	71.2%*
No	11.4%	0.6%	28.8%*

People in cluster 2 were mostly male (83.1%), many were Khmu (42.7%) and from Luang Prabang Province (70.2%). Sanitation and education levels were lower than the majority of cluster 1, however, the main differences were due to contact with pigs and consumption habits. Most were involved with pig-slaughtering (83.7%), handled offal and/or raw meat (99.6%) consumed raw pigs’ blood (76.4%), and more had pigs in the household compared to cluster 1 (36.0%). Around half had access to toilets (56.2%) and used protected water sources (51.1%). However, most washed their hands after using the toilet (83.1%) and boiled water before consumption (87.1%). Some cluster 3 participants also used unprotected water sources (34.7%) and only a third boiled their water before consumption. Only 7.2% of this cluster had toilet access and most people did not always wash their hands after using the toilet (61.9%). This cluster appeared to have the lowest level of education with 42.4% having no schooling.

### Risk factor analysis

For the analysis the odds of testing seropositive for the various pathogens for people in cluster 2 and 3 were compared to the odds of testing seropositive in in cluster 1 (protected water sources, boiled water, good hygiene practices and relatively low pig contact). These results are summarised in Table 3. Compared to cluster 1, people in cluster 2 (higher pig contact: particularly in terms of slaughtering, handling offal/raw meat and more likely to drink raw pigs’ blood with moderate hygiene practices, mostly Luang Prabang Province) and people in cluster 3 (unprotected water sources, poorer hygiene practices, pigs in household, mostly Savannakhet Province) had 0.52 (95% CI: 0.33 to 0.82) and 0.42 (95% CI: 0.28 to 0.61) times the odds of testing seropositive for *T*. *spiralis*, respectively. Therefore cluster 1 had the highest risk of this parasite. Clusters 2 and 3 had 2.18 (95% CI: 1.37 to 3.45) and 2.30 (95% CI: 1.58 to 3.33) times the odds of testing seropositive for HEV, compared to cluster 1, respectively. People in cluster 2 (high pig contact) were also more likely to test seropositive for JEV (OR: 2.49, 95% CI: 1.12 to 5.19) and cluster 3 (poor sanitation) were more likely to test seropositive for *Taenia* spp. (OR: 3.38, 95% CI: 1.12 to 10.2) and cysticercosis (OR: 2.69, 95% CI: 1.00 to 7.50), compared to cluster 1.

**Table 3:** Random-effects logistic regression analysis for association between cluster membership and testing seropositive for pig zoonoses.

Variables	OR (95% Confidence Interval)
***HEV***	
Cluster:	
1: Better sanitation, lower pig contact	1
2: Moderate sanitation, slaughter pigs	2.18 (1.37 to 3.45)
3: Poorer sanitation, moderate pig contact	2.30 (1.58 to 3.33)
Age category (baseline children):	3.36 (2.26 to 4.99)
Gender:	
Female	1
Male	1.34 (0.95 to 1.88)
***T*. *spiralis***	
Cluster:	
1: Better sanitation, lower pig contact	1
2: Moderate sanitation, slaughter pigs	0.52 (0.33 to 0.82)
3: Poorer sanitation, moderate pig contact	0.42 (0.28 to 0.61)
Age category (child baseline)	1.83 (1.27 to 2.65)
Gender:	
Female	1
Male	1.49 (1.06 to 2.10)
***Taenia spp*.**	
Cluster:	
1: Better sanitation, lower pig contact	1
2: Moderate sanitation, slaughter pigs	2.76 (0.78 to 9.72)
3: Poorer sanitation, moderate pig contact	3.38 (1.12 to 10.2)
Age category (child baseline)	1.60 (0.55 to 4.65)
***Cysticercosis***	
Cluster:	
1: Better sanitation, lower pig contact	1
2: Moderate sanitation, slaughter pigs	1.85 (0.55 to 6.23)
3: Poorer sanitation, moderate pig contact	2.69 (1.00 to 7.50)
Age category (child baseline)	0.92 (0.32 to 2.66)
***JEV***	
Cluster:	
1: Better sanitation, lower pig contact	1
2: Moderate sanitation, slaughter pigs	2.49 (1.12 to 5.19)
3: Poorer sanitation, moderate pig contact	1.18 (0.54 to 2.60)
Age category (child baseline)	1.20 (0.57 to 2.52)

Farmers that called an animal health worker (or similar) if their pig was sick had 0.38 (95% CI: 0.18 to 0.80) times the odds of having pigs test seropositive for *T*. *spiralis*, compared to farmers which reported self-treating their pigs (Table 4). Pigs kept in tethered systems had 0.85 (95% CI: 0.18 to 0.91) times the odds of testing seropositive for hepatitis E compared to those in penned systems. Further; households that disposed of pig manure in water sources had 2.39 (95% CI: 1.07 to 5.34) times the odds of testing seropositive for hepatitis E.

**Table 4:** Regression analysis of risk factors for pig seropositivity for *T*. *spiralis* and HEV.

Variable	OR (95% CI)
***T*. *spiralis***	
Action when pig is sick	
Self-treat	1
Call VA	0.38 (0.18 to 0.80)
Do nothing	1.12 (0.54 to 2.32)
Age (months)	1.02 (1.03 to 1.10)
**HEV**	
Housing (wet season)	
Penned	1
Tethered	0.85 (0.18 to 0.91)
Free range (at least sometimes)	0.40 (0.28 to 1.10)
Manure disposal	
Other (mostly pen)	1
Water	2.39 (1.07 to 5.34)
Fertilizer	0.59 (0.44 to 1.06)
Age (months)	1.07 (1.03 to 1.10)

## Discussion

### Hepatitis E

The seroprevalence of HEV in humans was very high, particularly in Savannakhet province. Seroprevalence does not necessarily indicate recent infection as humans may be exposed at a young age and develop immunity to subsequent exposures. However, it does suggest circulation of the virus in the area. Cluster 2 and 3 had increased odds of seropositivity for HEV compared to cluster 1. Presumably, the main transmission route is consumption of contaminated water as these clusters were more likely to use unprotected water sources and practice open defaecation. People in cluster 3 were also much less likely to boil water before consumption and wash their hands after.

The zoonotic nature of the disease was suggested as people in cluster 2 were more likely to have occupational contact (slaughtering and handling pigs). This has previously been reported as a risk factor for infection [11], although adult pigs are usually free of virus shedding. However, we cannot be sure that humans had a zoonotic strain of the virus as only two (genotype 3 and 4) of the four virus genotypes affecting humans are commonly found in pigs. In a previous study in Luang Prabang district 15.7% (95% CI: 5.4 to 26.0) of pigs had detectable HEV RNA (genotype 4) in their faeces [7]. In the current study, pigs from households where manure ended up in water sources were more likely to be seropositive for HEV. HEV strains of swine origin have previously been identified in surface water; which may also present an additional route of transmission to humans [26]. Hepatitis E is responsible for more than 50% of cases of acute hepatitis in endemic countries and the disease has a high case fatality rate in pregnant women [27].

### Trichinella

People in cluster 1 appeared to have the highest risk of *T*. *spiralis*. More people in this cluster reported eating fermented sausage (compared to cluster 2) and were from Luang Prabang Province (compared to cluster 3). Most previous outbreaks have been reported in the North [5]. Although cluster 1 had the best hygiene practices and higher education levels they appeared to have a higher risk of *T*. *spiralis*. However, due to its route of transmission (consumption of contaminated meat) it is generally associated with wealthier Lao residents who tend to consume meat more often [8]. Heavy parasite loads can lead to myocarditis, encephalitis or death. The International Commission on Trichinellosis recommends a range of measures including well-cooked pork and not feeding undercooked pork to pigs in swill.

### Taenia/cysticercosis

Cluster 3 had the highest risk of *Taenia* spp./cysticercosis and people in this cluster were the most likely to practice open defaecation (92.5%), which is one of the biggest risk factors for cysticercosis [16]. Around 50% of the cluster consumed fermented sausage which may lead to ingestion of the tapeworm (taeniasis); however, this was a similar figure to cluster 1 which had a lower risk of testing seropositive for *Taenia* spp. It is possible that in areas where open defaecation is practiced, ingestion of contaminated vegetation and/or water by pigs and humans is more likely, maintaining the parasite lifecycle [6]. In addition, cluster 3 were mainly from Savannakhet where more than half the pigs sampled were tethered or free grazed, compared to Luang Prabang Province where 90.6% were penned, suggesting that pigs may have greater access to human faecal matter in this province. Although it is assumed humans were infected with *T*. *solium*, *T*. *hydatigena* may also have been responsible for seropositive results.

Cysticercosis should be a priority disease for control as it is endemic throughout Southeast Asia, is the leading cause of epilepsy in the region, and is currently classified as a Neglected Tropical Disease [4]. Following this study an intervention was launched in a very high prevalence (“hyper-endemic”) village. This combined Mass Drug Administration in humans with vaccination and anthelmintic treatment in pigs. This was done in conjunction with education campaigns to increase community awareness and knowledge of the risks of the disease and preventive measures, in order to discourage open defaecation. This intervention has promising results to date [28]. The selection of the study areas aimed for a representative cross-section of villages in Lao PDR in terms of ethnicities and production systems. Therefore study findings may be generalised to some other areas. The results of this study could be used to inform entry points for intervention. For example targeting villages for risk-based surveillance and control activities such as reduction of open defaecation practices, particularly with high levels of pigs in free range scavenger systems.

### Japanese encephalitis

Japanese encephalitis is a major public health concern due to its’ high mortality and morbidity, particularly in younger children [27]. The percentage of seropositive pigs was 73.0% which is very similar to a previous study in Northern Lao PDR [9]. People in cluster 2, with the highest seroprevalence were mainly from Luang Prabang and had the highest pig contact. Pigs are a reservoir for human infection and per capita pig density is reported to be high in the northern region of Lao PDR [1]. A higher proportion of pigs were seropositive to the JEV MAC test in the North; this detects IgM antibodies indicating recent infection. Use of mosquito nets in the sampled villages was ubiquitous and it appears close pig contact poses the biggest risk in the region.

### Study limitations

The study has several limitations; the main limitation is that pigs and humans were recruited separately therefore correlation of infection within households cannot be investigated as part of this study. Robust seroprevalence estimates were achieved for the human part of the survey, but not for pigs. Adjusting the estimates to predict pig seroprevalence might have been possible by applying weightings to the villages according to their proportion of the total provincial pig population. However, village-level pig population data was not available, even at the Provincial level. Prevalence estimates based on serology do not give an accurate estimate of recent infections and risk factor analysis may have excluded past exposures. However, it does mean that past infections are included in the study. In addition, cross-reactivity for *Taenia* spp. can occur with other parasitic infections e.g. echinococcosis, schistosomiasis, angiostrongyliasis and fasciolasis [23, 29]. Recent high quality evidence on the presence of these parasites in the study areas is lacking, therefore the likelihood of false positive results cannot be assessed.

As risk factor analysis was performed using the results of the cluster analysis the risk factors are aggregated and associations with specific exposures and the pathogens are not explicitly assessed. However, many of the exposures were highly correlated and the seroprevalence of cysticercosis, *Taenia* spp. and JEV was very low and HEV seroprevalence very high which made multivariate risk factor analysis using traditional methods difficult. Despite these drawbacks the results provide useful information on the burden and routes of transmission of important zoonotic pathogens of pigs for a country where surveillance data is lacking.

### Recommendations

Meat inspection is recommended for the control of certain zoonoses including trichinellosis and taeniasis/cysticercosis but informal slaughter practices, lack of secure funding, limited technical capacity and limited data on the supply chain make this very difficult to implement in Lao PDR. Many farmers rely on middlemen who buy and sell their pigs and the point of slaughter is often unknown. Therefore, farmers require practical and low cost options for control. Increasing awareness of the financial impact of zoonotic diseases in pigs may motivate farmers’ to participate in disease control.

Over 80% of individuals in cluster 2, which had higher risk of *Taenia* spp., cysticercosis and HEV, did not wash hands after using the toilet or boil water before consumption. Furthermore, 42.3% of all individuals in the current study practiced open defaecation. Simple low-cost measures such as correct hand washing and reducing consumption of undercooked meat may reduce the zoonotic disease burden in Lao PDR. However, many of these factors are socio-cultural; encouraging behaviour change can be difficult and education campaigns are needed. Schools may provide a good starting point for education interventions, provided they are attended by the majority of children. School-led sanitation programs in other developing countries have shown some success in encouraging hand washing and reducing open defaecation near schools, and the community [30].

### Conclusions

This study highlights the importance of zoonotic diseases originating from pigs in Lao PDR and has identified typologies of individuals who are at higher risk of infection. Funding for disease control in Lao PDR is lacking therefore recommendations for realistic and low-cost disease control measures in both pigs and humans are required. Increasing disease awareness may motivate farmers to participate in disease control and encourage Laotians to use simple preventive measures to reduce transmission of these pathogens to humans.

## Supporting Information

S1 Checklist STROBE checklist. (DOC)
